# Simple risk scoring using sinus rhythm electrocardiograms predicts the incidence of atrial fibrillation in the general population

**DOI:** 10.1038/s41598-024-60219-y

**Published:** 2024-04-26

**Authors:** Hiroyuki Kamada, Shin Kawasoe, Takuro Kubozono, Yuichi Ninomiya, Kei Enokizono, Issei Yoshimoto, Yasuhisa Iriki, Yoshiyuki Ikeda, Masaaki Miyata, Hironori Miyahara, Koichi Tokushige, Mitsuru Ohishi

**Affiliations:** 1 Department of Cardiovascular Medicine and Hypertension, Kagoshima University Graduate School of Medical and Dental Sciences, 8-35-1 Sakuragaoka, Kagoshima, 890-8520 Japan; 2 Kagoshima Kouseiren Hospital, Kagoshima, Japan

**Keywords:** Cardiovascular diseases, Atrial fibrillation, Preventive medicine

## Abstract

Atrial fibrillation (AF) is an arrhythmic disease. Prediction of AF development in healthy individuals is important before serious complications occur. We aimed to develop a risk prediction score for future AF using participants’ data, including electrocardiogram (ECG) measurements and information such as age and sex. We included 88,907 Japanese participants, aged 30–69 years, who were randomly assigned to derivation and validation cohorts in a ratio of 1:1. We performed multivariate logistic regression analysis and obtained the standardised beta coefficient of relevant factors and assigned scores to them. We created a score based on prognostic factors for AF to predict its occurrence after five years and applied it to validation cohorts to assess its reproducibility. The risk score ranged from 0 to 17, consisting of age, sex, PR prolongation, QT corrected for heart rate prolongation, left ventricular hypertrophy, premature atrial contraction, and left axis deviation. The area under the curve was 0.75 for the derivation cohort and 0.73 for the validation cohort. The incidence of new-onset AF reached over 2% at 10 points of the risk score in both cohorts. Thus, in this study, we showed the possibility of predicting new-onset AF using ECG findings and simple information.

## Introduction

Atrial fibrillation (AF) is a common arrhythmic disease associated with serious illnesses, including stroke and heart failure, that can impact patients’ lives. Anticoagulation is important for patients at risk of stroke due to AF^1^. Anticoagulation lowers the risk of stroke in patients with AF and could prevent up to two thirds of strokes^2,3^. Additionally, rhythm control, including catheter ablation for early AF, reportedly improves life outcomes^4^. Detecting AF is challenging and often occurs after a stroke or other serious events^5^. Many patients with AF have both asymptomatic and symptomatic episodes, with up to 40% of patients being asymptomatic. Asymptomatic AF carries a similar risk of stroke and mortality to symptomatic AF^6^. Predicting the risk of developing AF before serious related complications occur in a healthy population is critical and challenging.

Developments based on clinical risk factors for AF have been made, such as the introduction of the Cohorts for Heart and Aging Research in Genomic Epidemiology for Atrial Fibrillation (CHARGE-AF) model and the Suita study^7–9^. The risk of developing AF is affected by patients’ genetic predispositions and clinical risk factors^10^. A risk score was reported for the CHARGE-AF score based on AF’s clinical risk factors and genetic predisposition data^11^. The possibility of developing AF in individuals with a high genetic risk score was considered higher than that in those with a low score, even if they had low CHARGE-AF scores. Moreover, a study has predicted the risk of developing AF using deep learning to apply artificial intelligence (AI) to electrocardiograms (ECGs)^12^. AI-based prediction of AF is also useful, but the cost of using AI and the nature of the decision criteria as a black box are problems encountered worldwide^13^. In this study, we investigated whether a simple risk model using 12-lead ECG could predict the risk of developing AF.

## Methods

### Study population

**Figure 1:**
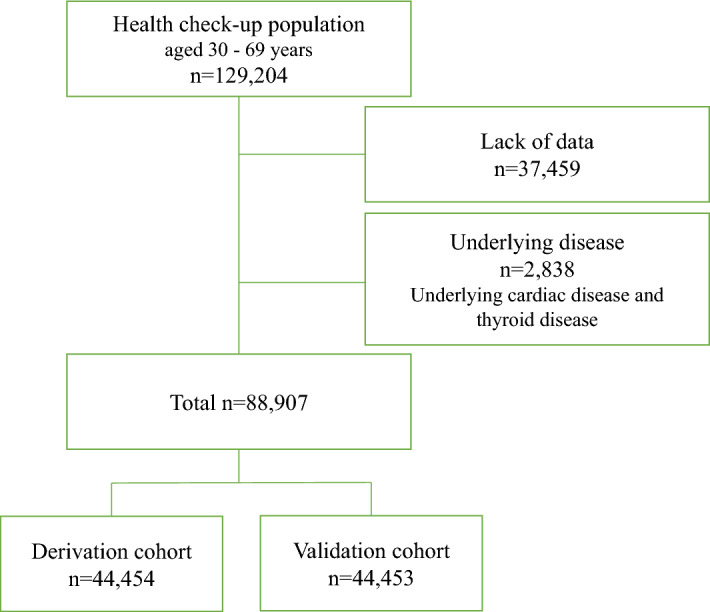
Flowchart of the study population. We enrolled 129,204 participants who underwent physical examinations and excluded 37,459 who lacked sufficient data and 2838 with underlying diseases. This study included 88,907 participants, aged 30–69 years, who were randomly assigned to derivation and validation cohorts at a ratio of 1:1.

The flowchart of this study is shown in Fig. 1. The inclusion criteria were individuals who underwent at least two annual physical examinations with available data at baseline and after 5 years, while the exclusion criteria were individuals without basic information (height, weight, history of smoking, history of alcohol intake, or underlying diseases) or ECG data. The present study aimed to examine the risk of AF in a general healthy population with no obvious underlying disease. Participants with thyroid disease or underlying heart disease with a definite risk of AF were excluded. We retrospectively enrolled 129,204 consecutive participants, aged 30–69 years, who underwent at least two annual physical examinations with available data at baseline and after 5 years (median, 5.0 years; 1st quartile, 4.4 years; 3rd quartile, 5.1 years) at the Kagoshima Kouseiren Hospital between January 1979 and December 2016. A total of 88,907 participants were examined, excluding 37,459 who lacked sufficient data and 2838 with underlying diseases (846, 2010, and 18 with underlying heart diseases [e.g. AF, valvular disease, cardiomyopathy, congenital heart disease, and ischemic heart disease], thyroid diseases, and coexisting heart and thyroid diseases, respectively). Of the participants who lacked sufficient data, 19,708 had no basic information (height, weight, history of smoking, history of alcohol intake, or underlying diseases), and 17,751 had no ECG data. The included participants were randomly assigned to derivation and validation cohorts at a ratio of 1:1. This study was approved by the institutional ethics committee of Kagoshima University Graduate School of Medical and Dental Sciences (approval no. 170130 [520], 4th August 2017). The need for informed consent was waived by the ethics committee because only existing anonymized data were used in this study. The study complied with the principles of the Declaration of Helsinki.

### Data collection

We obtained data on age, sex, height, and weight. Based on a median age of 52 years, participants were divided into two groups: aged ≤ 51 years and > 52 years. Height and weight were measured using standard anthropometric methods. The body mass index (BMI) was calculated for each participant. BMI was calculated as weight (kg) divided by height squared (m^2^), and participants were categorised into groups with BMIs of ≤ 24.9 kg/m^2^ and ≥ 25.0 kg/m^2^; obesity was defined based on the Japanese definition, BMI of ≥ 25.0 kg/m^2^^14^. Information on the history of smoking (currently smoking or history of smoking) and alcohol intake (drinking > 10 days per month) and underlying diseases (underlying cardiac and thyroid diseases) was obtained by questioning each participant at the time of the physical examination. These data were collected from each participant’s medical record. A 12-lead surface ECG was performed for each participant during routine annual physical examination. We used automatic measurements and discriminations of 12-lead ECGs. Heart rate (HR), QRS duration (QRSd), axis range, QT interval (QTi), SV1, and RV5 were obtained by automatic ECG measurement. PR prolongation (PR > 200 ms), premature atrial contraction (PAC), and premature ventricular complex (PVC) were obtained by automatic ECG discrimination^15,16^. Left axis deviation (LAD) and right axis deviation (RAD) were defined as − 30° to − 90° and + 90° to + 180°, respectively^17^. The QT corrected for HR (QTc) was calculated using Bazett’s correction formula (QTc = QT/RR^1/2^). Participants were also categorised based on QRSd, ≤ 119 ms and ≥ 120 ms; QTc, ≤ 439 ms and ≥ 440 ms; and SV1 + RV5, ≤ 3.4 mV and ≥ 3.5 ms. QRS prolongation, QTc prolongation, and left ventricular hypertrophy (LVH) were defined as a QRSd of ≥ 120 ms^18^, QTc of ≥ 440 ms^19^ and SV1 + RV5 of ≥ 3.5 mV^20^ respectively. New-onset AF was defined as diagnosis of AF using an ECG 5 years after baseline measurements and new cases of AF diagnosed at any point during the 5-year period. The new cases of AF were based on ECG during annual physical examinations (mean number of ECGs during follow-up per participant: 3.7 ± 1.1) or based on the new AF history obtained by questioning each participant at the annual physical examinations. Secondary AF such as that caused by surgery or trauma might be included in participants who had the new AF history obtained by questioning.

### Statistical analysis

Continuous variables (age, BMI, HR, QRSd, QTi, QTc, SV1, RV5, and SV1 + RV5) were presented as means ± standard deviations. Categorical variables (sex, obesity, smoking, alcoholic intake, PR prolongation, QRS prolongation, QTc prolongation, LVH, PAC, PVC, RAD, and LAD) were presented as proportions (percentages). Differences between the two groups (between the derivation and validation cohorts or between the new-onset AF and sinus rhythm [SR] groups) for continuous and categorical variables were analysed using the Student’s unpaired *t*-test and the χ^2^ test, respectively. Univariate and multivariate logistic regression analyses were applied to calculate the odds ratio (OR) and 95% confidence interval for AF incidence. Significant factors in the univariate analysis were selected as dichotomous variables rather than continuous variables for the multivariate analysis. Multivariate analysis was conducted with upper categorical variables such as LVH or QTc prolongation, not with continuous variables such as RV5, RV1 + RV5, QT interval, or QTc interval. Obesity was analysed using the Japanese definition of obesity, BMI of ≥ 25.0 kg/m^2^^14^. To create a risk score that predicts 5-year incidence of AF, the following scores related to standardised beta coefficients were assigned to each risk factor category for items that were significant in the multivariate analysis based on the methodology used in the Japan Epidemiology Collaboration on Occupational Health Study Group’s findings: 1, β = 0.01–0.20; 2, β = 0.21–0.80; 3, β = 0.81–1.20; 4, β = 1.21–2.20; 5, β > 2.20^21^. The discriminative performance of the score was assessed using the area under the curve (AUC) from the receiver operating characteristic (ROC) analysis. Cochran-Armitage trend tests were performed to examine the constant trend toward higher incidence of AF with an increasing risk score. We evaluated the calibration using calibration plots. All data analyses were performed using the JMP Pro version 17 software (SAS Institute Inc, Cary, NC, USA. https://www.jmp.com/en_us/software/predictive-analytics-software.html). A p value of < 0.05 was considered statistically significant.

## Results

### Baseline characteristics

The baseline characteristics of the study population in the derivation and validation cohorts are shown in Table 1. No significant differences were observed between derivation and validation cohorts for all the factors. In the whole study population, the mean age was 51 ± 10 years, with 40,216 males (45.2%). The mean BMI was 23.2 ± 3.2 kg/m^2^, and the percentages of participants with obesity, those with a history of smoking, and those with a history of alcohol intake were 26.0%, 34.9%, and 53.2%, respectively. Concerning the ECG characteristics, the mean HR was 65.6 ± 10.4 bpm; moreover, PR and QRS prolongations were observed in 819 and 3,504 participants (0.9% and 3.9%), respectively. The mean QTc interval and SV1 + RV5 were 402.8 ± 23.1 ms and 2.61 ± 0.79 mV, respectively. Additionally, QTc prolongation, LVH, PAC, and LAD were observed in 5141, 11,538, 856, and 1086 participants (5.8%, 13.0%, 1.0%, and 1.2%), respectively.

**Table 1:** Baseline characteristics.

	Derivation cohort (n = 44,454)	Validation cohort (n = 44,453)	p value
Age, years	51 ± 10	51 ± 10	0.883
Sex, male	20,015 (45.0)	20,201 (45.4)	0.209
BMI, kg/m^2^	23.2 ± 3.1	23.2 ± 3.2	0.773
Obesity, n	11,573 (26.0)	11,540 (26.0)	0.802
Smoker, n	15,387 (34.6)	15,652 (35.2)	0.062
Alcoholic intake, n	23,668 (53.2)	23,649 (53.2)	0.901
HR, bpm	65.6 ± 10.4	65.6 ± 10.4	0.314
PR prolongation, n	407 (0.9)	412 (0.9)	0.860
QRS width, ms	95.4 ± 13.4	95.6 ± 12.5	0.114
QRS prolongation, n	1719 (3.9)	1785 (4.0)	0.255
QT interval, ms	388.1 ± 30.9	388.2 ± 30.8	0.626
QTc interval, ms	402.9 ± 23.2	402.8 ± 23.1	0.522
QTc prolongation, n	2608 (5.9)	2533 (5.7)	0.282
SV1, mV	0.94 ± 0.42	0.94 ± 0.41	0.939
RV5, mV	1.67 ± 0.59	1.67 ± 0.59	0.937
SV1 + RV5, mV	2.61 ± 0.79	2.61 ± 0.79	0.922
LVH, n	5723 (12.9)	5815 (13.1)	0.358
PAC, n	432 (1.0)	424 (1.0)	0.734
PVC, n	539 (1.2)	522 (1.2)	0.600
RAD, n	703 (1.6)	718 (1.6)	0.688
LAD, n	572 (1.3)	514 (1.2)	0.077

Continuous variables are expressed as means ± standard deviations. Categorical variables are expressed as numbers of subjects and proportions (percentages). Obesity was defined as a BMI of ≥ 25 kg/m^2^. PR prolongation was defined as a PR interval of > 200 ms. QRS prolongation was defined as a QRS width of ≥ 120 ms. QTc prolongation was defined as a QTc interval of ≥ 440 ms. LVH was defined as a SV1 + RV5 of ≥ 3.5 mV.

*BMI* body mass index, *HR* heart rate, *LVH* left ventricular hypertrophy, *PAC* premature atrial complex, *PVC* premature ventricular complex, *RAD* right axis deviation, *LAD* left axis deviation, *QTc* mean QT corrected for HR.

### Incidence of AF

Among the included participants, new-onset AF was observed in 152 (0.3%) during the 5-year period. Comparisons between participants with new-onset AF (the AF group) and those with SR (the SR group) are shown in Table 2. Age and BMI were higher in the AF group than those in the SR group (age, 59 ± 8 years vs. 51 ± 10 years, p < 0.001; BMI, 23.8 ± 3.4 kg/m^2^ vs. 23.2 ± 3.1 kg/m^2^, p = 0.016). The percentages of males, smoking, and alcohol intake were higher in the AF group than those in the SR group (males, 73.0% vs. 44.9%, p < 0.001; smoking, 52.0% vs. 35.6%, p < 0.001; alcohol intake, 65.1% vs. 53.2%, p = 0.003). No significant difference was observed in the percentage of obesity between the two groups. Concerning ECG characteristics, no significant difference in HR was found between the two groups. The percentages of PR, QRS, and QTc prolongations were higher in the AF group than those in the SR group (PR prolongation, 4.0% vs. 0.9%, p < 0.001; QRS prolongation, 7.2% vs. 3.9%, p = 0.031; QTc prolongation, 10.5% vs. 5.9%, p = 0.014). The QTc interval and SV1 + RV5 were higher in the AF group than those in the SR group (QTc interval, 407.2 ± 27.5 ms vs. 402.9 ± 23.2 ms, p = 0.021; SV1 + RV5, 3.00 ± 1.00 mV vs. 2.61 ± 0.79 mV, p < 0.001). The percentages of LVH, PAC, PVC, and LAD were higher in the AF group than those in the SR group (LVH, 25.7% vs. 12.8%, p < 0.001; PAC, 7.2% vs. 1.0%, p < 0.001; PVC, 3.3% vs. 1.2%, p = 0.019; LAD, 4.6% vs. 1.3%, p < 0.001). No significant difference was observed in RAD between the two groups.

**Table 2:** Differences between the new-onset AF and SR groups.

	New-onset AF (n = 152)	Sinus rhythm (n = 44,302)	p value
Age, years	59 ± 8	51 ± 10	< 0.001
Sex, male	111 (73.0)	19,904 (44.9)	< 0.001
BMI, kg/m^2^	23.8 ± 3.4	23.2 ± 3.1	0.016
Obesity, n	43 (28.3)	11,530 (26.0)	0.526
Smoker, n	79 (52.0)	15,308 (35.6)	< 0.001
Alcoholic intake, n	99 (65.1)	23,568 (53.2)	0.003
HR, bpm	64.6 ± 10.6	65.6 ± 10.4	0.208
PR prolongation, n	6 (4.0)	401 (0.9)	< 0.001
QRS width, ms	97.4 ± 13.0	95.4 ± 13.4	0.066
QRS prolongation, n	11 (7.2)	1708 (3.9)	0.031
QT interval, ms	395.7 ± 36.0	388.0 ± 30.9	0.002
QTc interval, ms	407.2 ± 27.5	402.9 ± 23.2	0.021
QTc prolongation, n	16 (10.5)	2592 (5.9)	0.014
SV1, mV	0.99 ± 0.50	0.94 ± 0.42	0.135
RV5, mV	2.01 ± 0.73	1.67 ± 0.59	< 0.001
SV1 + RV5, mV	3.00 ± 1.00	2.61 ± 0.79	< 0.001
LVH, n	39 (25.7)	5684 (12.8)	< 0.001
PAC, n	11 (7.2)	421 (1.0)	< 0.001
PVC, n	5 (3.3)	534 (1.2)	0.019
RAD, n	0 (0.0)	703 (1.6)	0.118
LAD, n	7 (4.6)	565 (1.3)	< 0.001

Continuous variables are expressed as means ± standard deviations. Categorical variables are expressed as numbers of subjects and proportions (percentages). Obesity was defined as a BMI of ≥ 25 kg/m^2^. PR prolongation was defined as a PR interval of > 200 ms. QRS prolongation was defined as a QRS width of ≥ 120 ms. QTc prolongation was defined as a QTc interval of ≥ 440 ms. LVH was defined as a SV1 + RV5 of ≥ 3.5 mV.

*AF* atrial fibrillation, *BMI* body mass index, *HR* heart rate, *LVH* left ventricular hypertrophy, *PAC* premature atrial complex, *PVC* premature ventricular complex, *RAD* right axis deviation, *SR* sinus rhythm, *LAD* left axis deviation, *QTc* mean QT corrected for HR.

### Risk factors of AF incidence

The results of univariate and multivariate logistic regression analyses are shown in Table 3. Univariate analysis showed significant differences in age, sex, BMI, smoking, and alcohol intake between the two groups. Concerning ECG findings, no significant difference was observed in HR between the groups. However, significant differences were identified in PR, QRS, and QTc prolongations, and LVH, PAC, PVC, and LAD between the groups. In multivariate analysis, age of ≥ 52 years (OR 3.81, p < 0.001), male sex (OR 3.26, p < 0.001), PR prolongation (OR 2.60, p = 0.025), QTc prolongation (OR 1.96, p = 0.014), LVH (OR 1.64, p = 0.010), PAC (OR 5.75, p < 0.001), and LAD (OR 2.46, p = 0.026) were independent prognostic factors.

**Table 3:** Univariate and multivariate analyses for the incidence of AF.

	Univariate analysis			Multivariate analysis		
	OR	95% CI	p value	OR	95% CI	p value
Age, years	1.08	1.06–1.10	< 0.001			
Age > 52 years	4.09	2.73–6.14	< 0.001	3.81	2.52–5.77	< 0.001
Sex, male	3.32	2.31–4.75	< 0.001	3.26	1.96–5.40	< 0.001
BMI, kg/m^2^	1.06	1.01–1.11	0.018			
Obesity	1.12	0.79–1.60	0.526			
Smoker	2.05	1.49–2.82	< 0.001	1.18	0.80–1.74	0.411
Alcoholic intake	1.64	1.18–2.29	0.004	0.93	0.62–1.40	0.730
HR, bpm	0.99	0.97–1.01	0.201			
PR prolongation	5.00	1.98–10.24	< 0.001	2.60	1.13–6.00	0.025
QRS width, msec	1.00	1.00–1.01	0.067			
QRS prolongation	1.95	1.05–3.60	0.034	1.06	0.56–2.00	0.856
QT interval, ms	1.01	1.00–1.01	0.002			
QTc interval, ms	1.01	1.00–1.01	0.021			
QTc prolongation	1.89	1.13–3.18	0.016	1.96	1.14–3.35	0.014
SV1, mV	1.32	0.92–1.90	0.135			
RV5, mV	2.04	1.69–2.48	< 0.001			
SV1 + RV5, mV	1.63	1.40–1.90	< 0.001			
LVH	2.34	1.63–3.38	< 0.001	1.64	1.13–2.38	0.010
PAC	8.13	4.37–15.13	< 0.001	5.75	3.05–10.84	< 0.001
PVC	2.79	1.14–6.83	0.025	1.88	0.75–4.67	0.176
LAD	3.74	1.74–8.01	< 0.001	2.46	1.12–5.43	0.026

Obesity was defined as a BMI of ≥ 25 kg/m^2^. PR prolongation was defined as a PR interval of > 200 ms. QRS prolongation was defined as a QRS width of ≥ 120 ms. QTc prolongation was defined as a QTc interval of ≥ 440 ms. LVH was defined as a SV1 + RV5 of ≥ 3.5 mV.

*BMI* body mass index, *CI* confidence interval, *HR* heart rate, *LVH* left ventricular hypertrophy, *OR* odds ratio, *PAC* premature atrial complex, *PVC* premature ventricular complex, *RAD* right axis deviation, *LAD* left axis deviation, *QTc* mean QT corrected for HR.

### Simple risk scores for AF incidence

**Figure 2:**
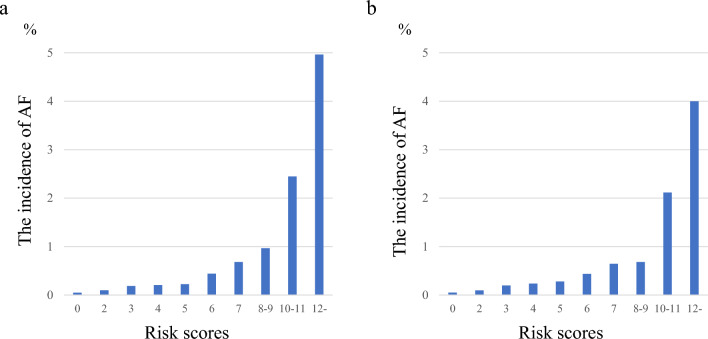
Incidence of AF in the simple predicting AF score. A higher simple predicting AF score correlated with a higher the incidence of AF ((**a**) derivation cohort, (**b**) validation cohort). *AF* atrial fibrillation.

**Figure 3:**
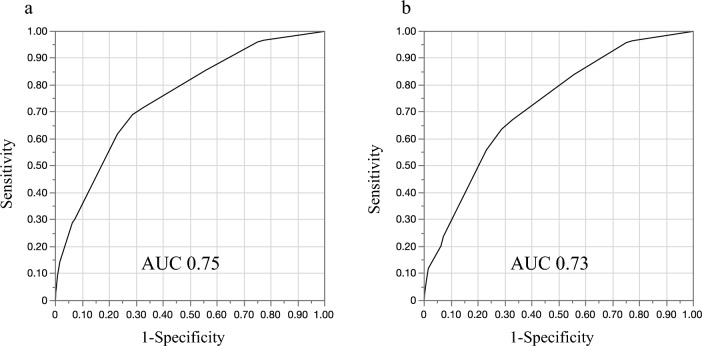
ROC curve for the incidence of AF and the simple predicting AF score. ROC curves were developed for the incidence of AF and the score in the derivation cohort (**a**) and validation cohort (**b**); the AUC was 0.75 and 0.73, respectively. *AF* atrial fibrillation, *AUC* area under the curve, *ROC* receiver operating characteristic.

**Figure 4:**
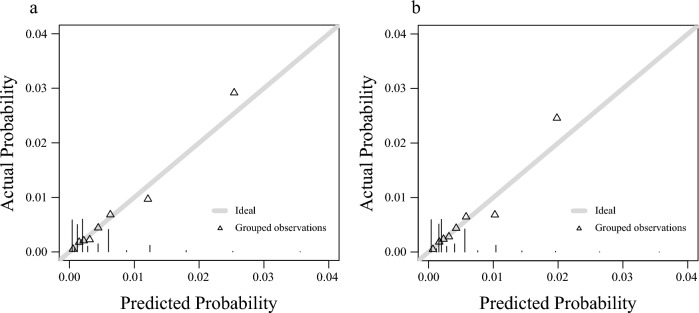
Calibration plots for the equation model in derivation and validation cohorts. The visual agreement between the AF predictions (predicted probability) and observations (Actual probability) for the equation model in the derivation cohort (**a**) and validation cohort (**b**). *AF* atrial fibrillation.

Standardised beta coefficients were calculated for the factors that were significant in the multivariate analysis (Table 4). Values for age (for those aged ≥ 52 years), sex (for males), PR prolongation, QTc prolongation, LVH, PAC, and LAD were 1.34, 1.18, 0.96, 0.67, 0.49, 1.75, and 0.90, respectively. Based on standardised beta coefficients, age (for those aged ≥ 52 years), sex (for males), PR prolongation, QTc prolongation, LVH, PAC, and LAD score were 4, 3, 3, 2, 2, 4, and 3, respectively. In the derivation cohort, the incidence of AF increased with increased simple predicting AF score (Fig. 2a). The incidence of AF reached ~ 0.4% at six points and > 2% at 10 points. The ROC curve for the discriminative ability of the generated scores to identify the incidence of AF is shown in Fig. 3a. The AUC was 0.75 (cut-off value of six points with a sensitivity and specificity of 69% and 71%, respectively). The SIMP_3_L_2_E AF risk score (Simple information [age, sex], PR interval, Prolongation of QTc, PAC, LVH, and LAD by ECG AF risk score) was applied to the validation cohort after confirming the results with the derivation cohort. In addition, the incidence of AF increased as SIMP_3_L_2_E predicting AF score increased (Fig. 2b). The ROC curve for the discriminative ability of the generated scores to identify the incidence of AF is shown in Fig. 3b. The AUC was 0.73 (cut-off value of six points with a sensitivity and specificity of 64% and 71%, respectively). Furthermore, the results adapted to the validation cohort were comparable to those of the derivation cohort. In the Cochran-Armitage trend test, both the derivation and validation cohorts were significant (derivation cohort, p < 0.001; validation cohort, p < 0.001), and a constant trend was identified in the incidence of AF with an increasing risk score. The results of the calibration are shown in Fig. 4. Good visual calibration is achieved for both the Derivation (Fig. 4a) and Validation cohorts (Fig. 4b).

**Table 4:** Standardised beta coefficients and assigned points for the risk factors.

Risk factors		β	Point
Age, years	30–51		0
	52–69	1.34	4
Sex, male	Women		0
	Men	1.18	3
PR prolongation	No		0
	Yes	0.96	3
QTc prolongation	No		0
	Yes	0.67	2
LVH	No		0
	Yes	0.49	2
PAC	No		0
	Yes	1.75	4
LAD	No		0
	Yes	0.90	3

PR prolongation was defined as a PR interval of > 200 ms. QTc prolongation was defined as a QTc interval of ≥ 440 ms. LVH was defined as a SV1 + RV5 of ≥ 3.5 mV.

*LVH* left ventricular hypertrophy, *PAC* premature atrial complex, *LAD* left axis deviation, *QTc* mean QT corrected for HR.

## Discussion

In this study, we showed the possibility of predicting new-onset AF using ECG findings and simple information, such as age and sex. Age of ≥ 52 years, male sex, PR prolongation, QTc prolongation, LVH, PAC, and LAD were independent prognostic factors and combined as the SIMP_3_L_2_E AF risk score. A higher calculated score using standardised beta coefficients correlated with a higher incidence of new-onset AF. The AUCs for the derivation and validation cohorts were 0.75 and 0.73, respectively. The incidence of new-onset AF reached > 2% at ten points of the risk score in both cohorts. To the best of our knowledge, this is the first report of a risk score based mainly on ECG to predict new-onset AF.

AF is the most common arrhythmia in clinical practice, and its incidence is rising globally^22^. It is a potentially health-threatening condition associated with an increased risk of ischaemic stroke, heart failure, cognitive impairment, and death; the presence of AF increases the risk of stroke five-fold^23^. Cerebral infarction due to AF thrombus is associated with a more extensive cerebral infarction than other types, with a significant impact on disability and mortality^24^. Furthermore, an estimated one-third of AF cases are asymptomatic, and stroke is not rare as a first symptom^5,25,26^. AF has been associated with sudden death, making detection and prediction of AF risk important in healthy individuals^27^. Age was also reported as a major risk factor for AF, and AF prevalence was higher with increasing age^23^. The prevalence of AF in males in the general United States population was 0.2%, 0.9%, 1.7%, 3.0%, 5.0%, 7.3%, 10.3%, and 11.1% for those aged < 55, 55–59, 60–64, 65–69, 70–74, 75–79, 80–84, and > 84 years, respectively. Additionally, the prevalence of AF for females was 0.1%, 0.4%, 1.0%, 1.7%, 3.4%, 5.0%, 7.2%, and 9.1% for those aged < 55, 55–59, 60–64, 65–69, 70–74, 75–79, 80–84, and > 84 years, respectively^28^. In contrast, in a previous study in Japan, the prevalence of AF for males in the general population was 0.2%, 0.8%, 1.9%, 3.4%, and 4.4% for those aged 40–49, 50–59, 60–69, 70–79, and > 79 years, respectively. Moreover, the prevalence of AF for females was 0.04%, 0.1%, 0.4%, 1.1%, and 2.2% for those aged 40–49, 50–59, 60–69, 70–79, and > 79 years, respectively^29^. The prevalence was higher in the United States for males and females than that in Japan. In the current study of participants aged 30–69 years, the prevalence of AF for males at baseline was 0.1%, 0.3%, 0.5%, and 1.3% for participants aged 30–39, 40–49, 50–59, and 60–69 years, respectively. The prevalence of AF for females at baseline was 0.05%, 0.05%, 0.2%, and 0.3% for those aged 30–39, 40–49, 50–59, and 60–69 years, respectively. The values representing the prevalence of AF in this study were comparable to those of the previous Japanese study^29^.

Age, sex, and hypertension are reportedly associated with risk factors for AF^30^. Furthermore, obesity and sleep disturbances are reportedly associated with the incidence of AF^31,32^. Meta-analyses showed that drinking and smoking habits were risk factors for AF^33–35^. Lifestyle-related diseases, such as hypertension, diabetes, and hyperuricemia, are also reported to be risk factors for AF. Moreover, scores using these risk factors have been reported to assess the risk of developing AF^7,8,30,36,37^. In this study, reviewing obesity, smoking habits, drinking habits, ECG findings, and simple information such as age and sex showed the possibility of predicting new-onset AF.

Several studies have examined risk factors for AF, which could be found using ECG. Interatrial, first-degree atrioventricular (AV), and right bundle branch blocks are reportedly risk factors for the incidence of AF^38–40^. ECG measurements and findings, such as PAC, p wave, and LVH, are reportedly risk factors for the incidence of AF^41^. Additionally, LAD has been related to the incidence of AF^41^. The prolonged QT interval has been reported to be associated with an increased risk of incident AF^42^. Recently, AI-based deep learning has been used to predict AF incidence^12,43,44^. In this study, after using general ECG measurements for evaluation, PR prolongation, QTc prolongation, LVH, PAC, and LAD were found to be independent predictors of the incidence of AF. Several AF risk scores for the incidence of AF have been previously proposed. The FHS clinical AF risk score was reported in 2009 with an AUC of 0.78 and validated with an AUC of 0.734 by Shulman et al. in 2016^30,45^. The ARIC AF risk score was reported in 2011 with an AUC of 0.765^46^. These AF risk scores include both clinical characteristics and ECG parameters. The FHS AF risk score included age, sex, other clinical characteristics, and ECG-based PR interval. The ARIC AF risk score included age, other clinical characteristics, and LVH, which was often measured with ECG. AF risk scores also have been demonstrated with AUCs of 0.716–0.765 by CHARGE-AF, HATCH, and C2HEST in studies by Alonso et al. in 2013, Suenari et al. in 2017, and Li et al. in 2019^7,47–49^. The HATCH and C2HEST scores were developed in Asian populations and did not use ECG parameters. To the best of our knowledge, no risk scores have been developed based mainly on ECG for predicting new-onset AF. The identified risk factors in this study were used to create a simple risk score using age, sex, and ECG measurements. The incidence of AF reached about 0.4% at six points and > 2% at ten points, and the AUCs for the derivation and validation cohorts were 0.75 and 0.73, respectively, in this study. The results were found to be comparable to previous risk scores. Although the cut-off value was six points, we considered that a cut-off value of ten points or more might be useful, as the incidence of AF increased frequently at that value. The risk score in this study demonstrated comparable predictive ability for AF in the general population using only ECG testing, in addition to clinical information such as age and sex. The advantage of this risk score is that it can predict AF using only existing ECG tests, without the need for complex tests or information. It might also be useful in elucidating the mechanism of ECG-based AF prediction using AI, which is problematic owing to the nature of its decision criteria as a black box.

This study had some limitations. First, this was a retrospective single-centre cohort study; thus, selective bias may have occurred. Therefore, another multicentre study or one with more cases than those used in our study is needed. Second, mechanical errors may have occurred in the automatic measurement and discrimination of ECGs. In addition, different machines were used, which may contribute to differences in discrimination owing to variations. The study was retrospective and could not include detailed ECG assessments, such as the shape and potential of the P wave, which could be important for the prediction of AF. Because it was not possible to examine the information about details at the time of AF, secondary AF such as that resulting from surgery or trauma might be included. Our data were collected over a long time period, and all available data was used to ensure the largest possible number of events. However, lifestyle changes throughout the time period may affected dietary habits and frequency of illnesses, and the use of outdated and inconsistent ECG machines may have affected the results. Prospective studies are needed to resolve these issues. Third, not all instances of AF may be have been detected. To solve this problem, improving evaluation methods using smart watches and long-term Holter ECGs is needed. Lastly, this study was performed on the general population; therefore, the low rate of AF incidence was considered a limitation. As the physical examinations in this study were conducted in participants aged < 70 years, no data were available for the older population, aged > 70 years, with a higher prevalence of AF. Therefore, obtaining data from a larger number of older individuals with a higher incidence of AF than those used in our study is necessary.

In this study, age of ≥ 52, male sex, PR prolongation, QTc prolongation, LVH, PAC, and LAD were independent prognostic factors for AF. We demonstrated the possibility of predicting new-onset AF using ECG findings and simple information such as age and sex. We did this by developing a simple score that does not require advanced techniques. Furthermore, owing to existing ECG tests, this methodology may be easily used in many hospitals and clinics. Notably, a large prospective study using improved evaluation methods to analyse data from a larger number of older individuals from the general population will help validate our results.

## Data Availability

The Kagoshima University Institutional Review Board and Kagoshima Kouseirin Hospital applied the restriction for public data sharing due to ethical and legal restrictions of the annual health check-up data containing sensitive information and that participants did not consent to public sharing. The deidentified data may be partly available upon ethical approval by request directed to Dr. Takuro Kubozono (kubozono@m.kufm.kagoshima-u.ac.jp).
